# Immunotherapy for recurrent glioblastoma: practical insights and challenging prospects

**DOI:** 10.1038/s41419-021-03568-0

**Published:** 2021-03-19

**Authors:** Xin Wang, Jie Lu, Gaochao Guo, Jinming Yu

**Affiliations:** 1 Departmenlt of Oncology, Renmin Hospital of Wuhan University, Wuhan, 430060 Hubei Province China; 2 Department of Radiation Oncology, Shandong Cancer Hospital and Institute, Shandong First Medical University and Shandong Academy of Medical Sciences, Jinan, 250117 Shandong Province China; 3 Department of Neurosurgery, The First Affiliated Hospital of Shandong First Medical University & Shandong Provincial Qianfoshan Hospital, Shandong Medicine and Health Key Laboratory of Neurosurgery, Jinan, 250117 Shandong Province China; 4 Department of Neurosurgery, Henan Provincial People’s Hospital, Cerebrovascular Disease Hospital, People’s Hospital Zhengzhou University, People’s Hospital Henan University, Zhengzhou, 450003 Henan China

**Keywords:** Cancer, CNS cancer

## Abstract

Glioblastoma (GB) is the most common high-grade intracranial malignant tumor with highly malignant biological behavior and a high recurrence rate. Although anti-PD-1/PD-L1 antibodies have achieved significant survival benefits in several kinds of solid tumors, the phase III clinical trial Checkmate 143 demonstrated that nivolumab, which targets PD-1, did not achieve survival benefits compared with bevacizumab in recurrent glioblastoma (rGB) patients. Nevertheless, neoadjuvant anti-PD-1 therapy followed by surgery and adjuvant anti-PD-1 therapy could effectively activate local and systemic immune responses and significantly improve the OS of rGB patients. Furthermore, several studies have also confirmed the progress made in applying tumor-specific peptide vaccination or chimeric antigen receptor-T (CAR-T) cell therapy to treat rGB patients, and successes with antibodies targeting other inhibitory checkpoints or costimulatory molecules have also been reported. These successes inspired us to explore candidate combination treatments based on anti-PD-1/PD-L1 antibodies. However, effective predictive biomarkers for clinical efficacy are urgently needed to avoid economic waste and treatment delay. Attempts to prolong the CAR-T cell lifespan and increase T cell infiltration through engineering techniques are addressing the challenge of strengthening T cell function. In this review, we describe the immunosuppressive molecular characteristics of rGB; clinical trials exploring anti-PD-1/PD-L1 therapy, tumor-specific peptide vaccination, and CAR-T cell therapy; candidate combination strategies; and issues related to strengthening T cell function.

## Facts

- Glioblastoma is a well-known “cold tumor” and has an immunosuppressive microenvironment. rGB has low immunogenicity and strong heterogeneity.
- Anti-PD-1/PD-L1 therapy has not achieved significant benefits in rGB compared with bevacizumab. However, neoadjuvant anti-PD-L1 therapy achieved survival benefits in a small cohort.
- Vaccination and CAR-T cell therapy in rGB has not achieved significant benefits in clinical trials.

## Open questions

- How to find more effective immune targets for tumor vaccine, immune checkpoint, and CAR-T therapy?
- How to select optimal combination strategies to overcome immunosuppressive factors?
- How to explore effective predictive biomarkers for immunotherapy in rGB to avoid economic waste and treatment delay?
- What method can open blood–brain barrier to improve drug permeability, and increase effective cytotoxic T lymphocyte cells?

## Introduction

Glioblastoma (GB) has an incidence of 0.59–3.69/100,000 people worldwide, with a median onset age of 63.0 years. The age-adjusted morbidity is 3.97/100,000 for males and 2.53/100,000 for females^1–3^. GB is a high-grade malignant brain tumor with characteristics of aggressive biological behavior and resistance to treatment. Even with treatment involving a combination of surgical resection, concurrent chemoradiotherapy, and adjuvant chemotherapy, more than 90% of GB patients will experience recurrence and progression. The median overall survival (OS) time of primary GB patients is 12–15 months, and the 5-year survival rate is only 9.8%^4,5^. In recent years, newly developed tumor treating fields (ttfields) have improved the median survival time of glioblastoma, but the time still remains less than 20 months^6^. Moreover, the median OS time of rGB is only 6–11 months owing to the lack of effective treatments^7–9^. GB is a highly vascularized tumor and expresses a large amount of vascular endothelial growth factor (VEGF)^10^. As a humanized monoclonal antibody to VEGF, bevacizumab (BEV) was approved by the FDA as the standard treatment for rGB to reduce the blood flow and volume of tumors^11,12^. However, BEV can remodel tumor blood vessels and lead to vascular malformations, which make tumors more hypoxic and resistant to treatment^13^.

Immune checkpoint blockade can inhibit signaling by the programmed death-1/programmed death-ligand 1 (PD-1/PD-L1) pathway, which downregulates cytotoxic CD8+ T cell activation and induces CD8+ T cell exhaustion^14^. With growing evidence supporting the efficacy of anti-PD-1/PD-L1 therapy, there have been obvious successes in advanced non-small cell lung cancer, renal cancer, chronic Hodgkin’s lymphoma, gastric cancer, urothelial cancer, cervical cancer, head and neck squamous cell carcinoma, hepatocellular carcinoma, and melanoma^15–19^, and several studies have been conducted to explore the clinical efficacy of checkpoint inhibitors in rGB. However, a phase III clinical trial demonstrated that nivolumab, which targets PD-1, did not produce survival benefits in patients with rGB compared with BEV^20^. Nevertheless, anti-PD-1 therapy combined with surgical treatment to reduce the tumor burden seems to be effective in activating local and systemic immune responses to rGB^21^. Although the clinical efficacy of anti-PD-1/PD-L1 therapy is controversial, checkpoint blockade therapy is still worth exploring in rGB. In addition to checkpoint blockade, tumor-specific peptide vaccination and CAR-T cell immunotherapy have also been explored in the treatment of rGB. Although progress has been made to date, given the antigenic heterogeneity and antigen escape of rGB, the path to achieving immunotherapeutic efficacy in rGB remains both bright and tortuous. In this review, we described the molecular characteristics of the rGB microenvironment and focused on the progress, potential combination strategies, and challenges of immunotherapy in rGB.

### Molecular features and tumor microenvironment of rGB

**Fig. 1:**
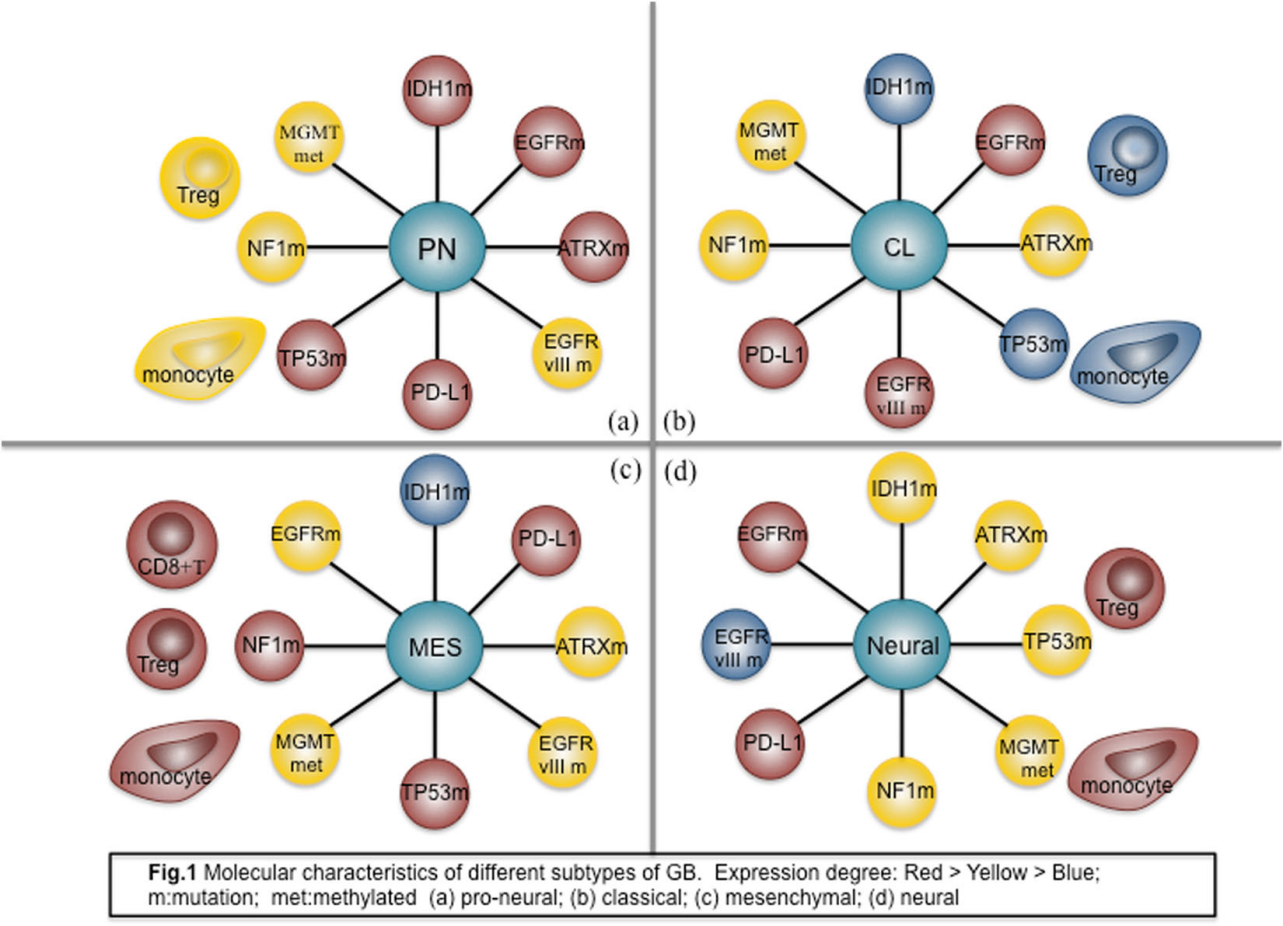
Molecular characteristics of different subtypes of GB. **a** pro-neural (PN) subtype; **b** classical (CL) subtype; **c** mesenchymal (MES) subtype; **d** neural subtype. Expression degree: Red > Yellow > Blue. m: mutation.

GB has been divided into four major subtypes based on genomic discrepancies: (1) neural, (2) pro-neural (PN), (3) classical (CL), and (4) mesenchymal (MES)^22^. These four subtypes have different gene alteration distributions, which could lead to distinct personal therapeutic strategies. Furthermore, several studies have found that different gene subtypes and gene alteration distributions exhibit diverse immune states in the tumor microenvironment (Fig. 1). For instance, Li et al.^23^ found that rGB has a higher IDH-1 mutation rate than primary GB and IDH-1 mutation occurs more frequently in the PN subtype. IDH-1 mutation in tumors usually indicates a relatively good prognosis, as shown by decreased expression of monocyte and regulatory T cell (Treg) markers in the tumor microenvironment and reduced expression of immune checkpoint receptors, which significantly attenuates the immunosuppressive effects of rGB on cytotoxic T cells^24–26^. In general, tumor-infiltrating lymphocytes (TILs) are depleted in the CL subtype, whereas they are enriched in the MES subtype^27^. In addition, ATRX positivity is common in the PN subtype, and a TCGA analysis demonstrated that ATRX-positive tumors had relatively few CD3+ and CD8+ T cells, which usually indicates a dismal prognosis^22^. Several studies have also illustrated the positive correlation between CD3+ and CD8+ T cell counts or infiltration in tumor tissues and therapeutic effects or prognostic outcomes^28–30^. In addition, TP53 mutation mostly occurs in the PN and MES subtypes and is relatively likely to be associated with the expression of the immune checkpoint receptors cytotoxic T lymphocyte-associated antigen-4 (CTLA-4) and PD-L1, which in turn affect T cell function^22,24,31^. However, no significant difference in the distribution of MGMT promoter methylation has been identified among the four major subtypes. MGMT promoter-methylated tumors commonly show decreased expression of CD8 and CD68 expression, which are immunobiological markers for T cells and macrophages, respectively^24^.

**Fig. 2:**
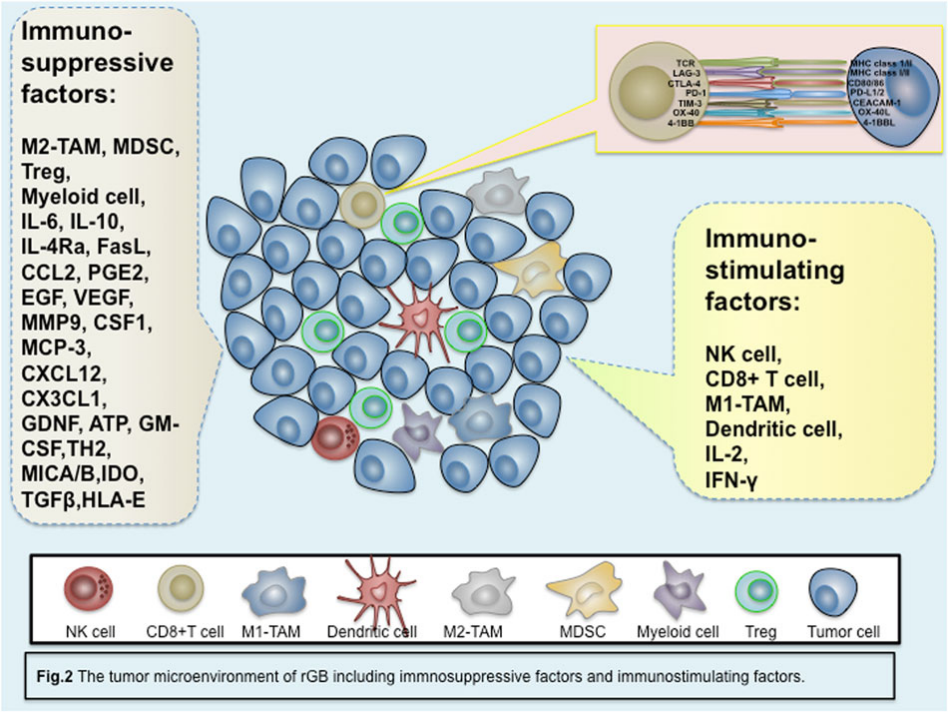
The tumor microenvironment of rGB. The immunosuppressive microenvironment of rGB is composed of a variety of immunosuppressive cells and cytokines which outweigh the role of immunostimulatory factors.

In addition to the biological properties of the tumor itself, a complex immune network contributes to the regulation of the biological behavior of the tumor. In the balance between immunostimulatory factors and immunosuppressive factors, immunosuppressive factors create obvious advantages by regulating the crosstalk between various cytokines and immune cells in rGB (Fig. 2). The immunostimulatory factors include effector immune cells including CD4+ T cells, CD8+ T cells, NK cells, and tumor-inhibiting M1 tumor-associated macrophages (M1-TAMs)^32–35^. However, the functions of these cells are usually exhausted and suppressed by immunosuppressive cells such as Tregs, tumorigenic M2-TAMs, myeloid cells, and MDSCs^36,37^. M2-TAMs, myeloid cells, and MDSCs can secrete various cytokines and factors, including IL-6, IL-10, IL-4Ra, FasL CCL2, PGE2, EGF, VEGF, and MMP9, to suppress cytotoxic T lymphocyte function^36,38–42^. Furthermore, T cell function is also suppressed by reducing IL-2 levels^43^ or IFN-γ levels^44,45^ and enhancing T_H_2 responses, which are regulated by Tregs^46^. In addition, tumor cells can inhibit T cell and NK cell activity by secreting MICA/B, IL-10, TGF-β, and HLA-E to recruit Tregs. GB is a poorly immunogenic cancer with increased PD-L1^47^, IDO^48^, and STAT3^49^ expression, while MHC^50^, costimulatory molecule^51^, and PTEN^52^ expression are reduced to recruit Tregs. Thus, Tregs play a critical role in the immunosuppressive tumor microenvironment.

### PD-1/PD-L1 checkpoint blockade in rGB

Several studies have demonstrated that the PD-1/PD-L1 pathway inhibits effector T cell function related to eradicating tumor cells^53–55^. In general, the clinical efficacy of anti-PD-1/PD-L1 therapy is positively correlated with the degree of PD-L1 expression in tumors^56–58^. However, the clinical efficacy of anti-PD-1/PD-L1 therapy is unpredictable in rGB. Berghoff et al.^31^ found that PD-L1 was expressed in 72.2% of rGB specimens. Zhao et al.^59^ carried out a retrospective study to explore the immune and genomic correlations of clinical response to anti-PD-1 therapy in 66 rGB patients. The 17 long-term responders exhibited enrichment in MAPK pathway alterations (PTPN11 and BRAF), and the 49 nonresponders showed obvious enrichment of PTEN mutations correlated with immunosuppression. These studies suggest that the clinical efficacy of anti-PD-1/PD-L1 therapy may be correlated with specific molecular alterations. Furthermore, a phase III clinical trial, Checkmate 143, demonstrated that in rGB patients who had previously received chemotherapy and radiation, an anti-PD-1 antibody (nivolumab) did not improve OS compared with BEV. The median OS time was 9.8 m for nivolumab vs. 10.0 m for BEV (Table 1)^20^. Thus, the results of clinical trials in rGB are disappointing. However, we are looking forward to a breakthrough in large-scale clinical trials of frontline treatment.

**Table 1:** Clinical trials of PD-1/PD-L1 checkpoint blockades in recurrent GB.

Trials	No.	Arms	Characteristic	Target	Phase	Results
NCT02550249	30	Nivo+continued surgery	Primary and recurrent GB	PD-1	II	mPFS:4.1 m; mOS:7.3 m;
NCT02852655	35	Pem+surgery+Pem	Recurrent/Progressive GB	PD-1	I	mPFS:2.4 m; mOS:13.7 m;
NCT02054806	26	Pem	PD-L1 expression≥1%	PD-1	I	mPFS:2.8 m; mOS:14.4 m; G3-4 TRAEs:15.4%
NCT02336165	159	Arm A: MEDI4736 + RT; Arm B: MEDI4736; Arm B2: MEDI4736 + Bev (10 mg/Kg); Arm B3: MEDI4736 + Bev (3 mg/Kg); Arm C: MEDI4736 + Bev	Arm A: unmethylated MGMT Arm B: Bev-naïve Arm B2: Bev-naïve Arm B3: Bev-naïve Arm C: Bev-refractory	PD-L1/VEGF	II	ArmB:6m-PFS:20%; 12m-OS:44.4%; G3-4 TRAEs:9.7%; ArmC: OS ≥ 22week: 36%; PFS ≥ 8weeks: 50%; G3-4 TRAEs: 4.5%
NCT02337491	80	Pem+Bev vs Pem		PD-1/VEGF	II	Safety; mOS: 6.8 m
NCT02017717	369 40	Nivo vs. Bev Nivo vs. Nivo+Ipi (Nivo3mg = 10; Nivo1mg + Ipi3mg = 10; Nivo3mg + Ipi1mg = 20)		PD-1/CTLA-4/VEGF	III I	mPFS: 1.5 m vs 3.5 m; mOS: 9.8 m vs 10.0 m; ORR: 8% vs 23%; G3-4 TRAEs 18% vs 15% Safety; Nivo3mg better Tolerated than other combinations 12m-OS: Nivo3mg: 40%; Nivo1mg + Ipi3mg: 30%; Nivo3mg + Ipi1mg: 35%
NCT02866747	62	HFSRT vs. HFSRT + Dur	Recurrent GB	PD-L1	I/II	NA
	20	Pem/Nivo+RT	Recurrent high-grade gliomas	PD-1		mPFS:4 m; mOS:10 m; ORR:35%;
NCT02794883	36	Dur vs. Tre+Dur		PD-L1/CTLA-4	II	NA
NCT02658981	100	Arm A1: Anti-LAG-3; Arm A2: Anti-CD137; Arm B1: Anti-LAG3 + Nivo; Arm B2: Anti-CD137 + Nivo		PD-1/LAG-3/CD137	I	NA
NCT02335918	175	Var+Nivo		PD-1/CD27	II	NA
NCT02798406	48	DNX-2401+ Pem	Recurrent GB and GS	PD-1	II	NA
NCT02648633	4	Valproate+SRS + Nivo	Recurrent GB	PD-1	I	NA
NCT02829931	26	HFSRT + Ipi+Nivo+Bev	Recurrent high-grade gliomas	PD-1/CTLA-4/VEGF	I	NA
NCT02658279	44	Pem	Hypermutator phenotype	PD-1	NA	NA
NCT02968940	43	Ave+HFRT	IDH mutant GB		II	NA
NCT02311582	58	Pem+MLA vs. Pem		PD-1	I	NA
NCT02430363	58	Pem vs. inhibitors of PI3K/Akt pathway		PD-1	I/II	NA
NCT02337686	18	Pem +surgery+Pem	Recurrent GB	PD-1	II	NA
NCT02529072	7	Arm A: Nivo+surgery+Nivo and DC vaccine; Arm B: Nivo and DC vaccine+surgery + Nivo and DC vaccine	Recurrent high-grade gliomas	PD-1	I	NA
NCT03493932	15	Anti-LAG-3+Anti-PD-1	Recurrent GB	LAG-3/PD-1	I	No significant survival difference between responders and nonresponders
NCT03532295	55	Anti-IDO1 + Anti-PD-L1 + RT	Recurrent GB	IDO1/PD-L1	I/II	NA
NCT03665545	24	Pem+IMA950	Recurrent GB	PD-1/multi peptide	I/II	NA
NCT03661723	60	Pem+RT	Recurrent GB Arm A: Bev-naïve Arm B: Bev-refractory	PD-1	II	NA
NCT03743662	94	Nivo+Bev+RT	Recurrent MGMT Methylated GB	PD-1	II	NA
NCT03233152	6	Ipi+Nivo	Recurrent GB	CTLA-4/PD-1	I	NA
NCT03707457	30	Nivo	Recurrent GB	PD-1	I	NA
NCT03341806	30	Ave+Laser Interstitial Therapy	Recurrent GB	PD-L1	I	NA
NCT03291314	52	Ave+Axitinib	Recurrent GB	PD-L1/VEGFR	II	NA
NCT03430791	60	TTF + Nivo+Ipi	Recurrent GB	PD-1/CTLA-4	II	NA

*GB* glioblastoma, *GS* gliosarcoma, *Nivo:Nivolumab* anti-PD-1 antibody, *Pem:Pembrolizumab* anti-PD-1 antibody, *TMZ* temozolomide, *Ave:Avelumab* anti-PD-L1 antibody, *PD-1-PIK T cells* pluripotent immune killer T cells express PD-1 antibody, *HFRT* hypofractionated radiation therapy, IDH isocitrate dehydrogenase, *MLA* MRI-guided laser ablation, *Ipi:Ipilimumab* anti-CTLA-4 antibody, *VEGF* vascular endothelial growth factor, *Tre* Tremelimumab, Anti-CTLA-4 antibody, *Dur: Durvalumab* anti-PD-L1 antibody, *Var* Varlilumab, Anti-CD27 antibody, *OVT* oncolytic virotherapy, *HFSRT* hypofractionated stereotactic irradiation, *Anti-PD-L1 CSR T cells*: autologous chimeric switch receptor engineered T cells redirected to PD-L1, *DNX-2401* a genetically modified oncolytic adenovirus, *DC* dendritic cell, *HSPPC-96* a vaccine made from fresh tumor taken at the time of surgery, *DCVax-L* autologous DC pulsed with tumor lysate antigen vaccine, *Cabiralizumab* Anti-CSF-1R antibody, *IMA950* novel multi-peptide therapeutic vaccine, *Axitinib* anti-VEGFR, *TTF* tumor treating field.

Wherry et al. demonstrated that TILs highly express PD-L1, CTLA-4, lymphocyte activation gene-3 (LAG3), CD95, PD-1, and T cell immunoglobulin domain and mucin domain-3 (TIM-3), which leads to T cell exhaustion^60^. Furthermore, exhausted CD8+ cytolytic T lymphocytes (CTLs) exhibit a PD-1+/TIM-3+ phenotype in tumors and induce adaptive resistance to anti-PD-1/PD-L1 therapy^61,62^. Considering the multiple immunosuppressive mechanisms observed in GB, a combination of several immunomodulators may be required to achieve the best therapeutic effect. For instance, activation of specific costimulatory receptors (such as OX40) and blockade of specific coinhibitory receptors (such as PD-1 or CTLA-4) could reduce tumor volume and prolong survival time in glioma animals models^32,63,64^. Other candidate checkpoint molecules that may be effectively targeted include OX40 and LAG3. Although immune checkpoint blockade combination therapy has achieved promising effects in preclinical GB models, the efficacy in clinical trials needs to be further verified.

Cloughesy et al^21^. evaluated the immunoreactivity and survival of 35 surgically resectable rGB patients following neoadjuvant and/or adjuvant therapy with pembrolizumab and found that the cohort treated with neoadjuvant anti-PD-1 therapy had significantly improved OS compared to the cohort without neoadjuvant anti-PD-1 therapy (*P* < 0.05). They also reported that neoadjuvant anti-PD-1 therapy resulted in increased expression of T cell- and interferon-γ-related genes and reduced the expression of cell cycle-related genes within tumors. Simultaneously, focal induction of PD-L1 and increased clonal expansion of T cells in the tumor microenvironment decreased PD-1 expression on peripheral T cells and a decreased monocytic population was more common in the neoadjuvant cohort. The median OS time of the neoadjuvant cohort was 13.7 m, whereas that of the adjuvant-only cohort was 7.5 m.

By comparing the results of these two clinical trials^20,21^, we can easily conclude that it is difficult to reverse the immunosuppressive tumor microenvironment of rGB with a single anti-PD-1 antibody. Nevertheless, when the tumor burden is reduced by surgery and combined neoadjuvant therapy and adjuvant PD-1 checkpoint blockade are administered, the “sandwich treatment strategy” can effectively activate local and systemic immune responses and significantly improve the OS of rGB patients. Schalper and colleagues confirmed that the underlying mechanism by which neoadjuvant anti-PD-1 therapy plays a critical role may involve increased chemokine transcript expression, enhanced infiltration, and T cell receptor (TCR) clonal diversity in the effector T lymphocyte population. They also demonstrated that nivolumab did not change the immune cell distribution in the GB microenvironment, based on an immunofluorescence assay comparing before and after therapy. Similarly, T cell function discrepancies were not found when comparing before and after nivolumab treatment^65^. However, the control group of GB patients showed reductions in lymphoid and myeloid cell numbers during the disease course, which demonstrated that nivolumab could maintain T cells in the tumor microenvironment.

### The current state of rGB vaccination

Immunotherapeutic strategies have focused on triggering specific immune responses targeting tumor-associated antigens (TAAs). GB-associated TAAs include CD133, YKL-40, gp100, epidermal growth factor receptor vIII (EGFRvIII), Wilms’ tumor 1 (WT1), IL-4, survivin, IL-13Rα2, HER2, and erythropoietin-producing hepatocellular receptor tyrosine kinase class A2 (EphA2)^66–70^. rGB is highly heterogeneous, and studies show that vaccines targeting only one tumor antigen have difficulty achieving optimal clinical effects unless the antigen is widely expressed in tumor cells. Desjardins et al. conducted a study of rGB patients and evaluated the clinical effect of a recombinant nonpathogenic polio-rhinovirus chimera (PVSRIPO), which induces recognition of the poliovirus receptor CD155 that is widely expressed in solid tumors and the tumor microenvironment. The OS of patients receiving PVSRIPO reached a plateau of 21% at 24 months that was sustained at 36 months^71^. In addition, Bloch et al. investigated the clinical efficacy and safety of heat-shock protein peptide complex-96 (HSPPC-96) vaccination in rGB patients following surgical resection and reported that the median OS time was 42.6 weeks (Table 2). However, they demonstrated that the patients with pretreatment lymphopenia had shorter OS than those without lymphopenia^72^. Thus, we can conclude that selecting pretreatment lymphopenia as a screening condition for improving clinical efficacy may be a good tactic. A phase II clinical study was also conducted to investigate the clinical responses to WT1 vaccination in rGB patients with human leukocyte antigen (HLA)-A24 positivity^73^. However, the results were limited. The overall response rate was 9.5%, and the median progression-free survival (PFS) time was 20.0 weeks. Similarly, Sakai et al. performed immunomonitoring of rGB patients treated with WT1-pulsed DC vaccination therapy. After the final vaccination, all rGB patients showed PD. The OS of the six rGB patients included in the study ranged from 4 to 13 months^74^. The researchers that found DC-based vaccination could induce and activate tumor antigen-specific CTLs, whereas WT1 peptide vaccination therapy could not increase WT1-specific CTL numbers in patients with rGB^73^, which fully demonstrated that DCs play a crucial role in immune regulation and that tumor-specific peptide-pulsed DC vaccination prolongs rGB patient survival. Thus, we cannot ignore the role of DCs as immune-boosting adjuvants in vaccination therapy^74,75^. With regard to DC-based immunotherapy, Vleeschouwer et al. also investigated the therapeutic effect of adjuvant vaccination on rGB patients. The median OS time was 9.6 months, with a 2-year OS rate of 14.8%. Therefore, adjuvant DC-based immunotherapy in rGB patients could induce long-term survival^76–78^. Although the superiority of tumor-specific peptide-pulsed DC vaccination therapy in rGB patients has been confirmed, it takes several months for autologous DCs to be isolated and purified; taking into account that rGB is a rapidly progressing disease, there are still many difficulties that need to be overcome. Furthermore, clinical studies have explored the immunogenicity of vaccination with synthetic TAA peptides. Okada et al. evaluated vaccination with α-type 1 polarized DCs (αDC1) loaded with synthetic peptides derived from EphA2, IL-13Rα2, YKL-40, and gp100 in rGB patients. Positive immune responses were found in 58% of the patients, demonstrating obvious increases in the levels of type 1 cytokines and chemokines. Above all, one rGB patient showed a sustained complete response^79^. Furthermore, a randomized phase III clinical trial evaluating personalized peptide vaccination (PPV) in human leukocyte antigen (HLA)-A24 + rGB patients was conducted, but neither the primary endpoint (OS) nor the secondary endpoint was reached^80^. Based on the above information, vaccination can be considered an effective approach to improve the survival time of rGB patients, although the negative results of a current phase II/III clinical trials have challenged vaccination as single-mode immunotherapy.

**Table 2:** Clinical trials of tumor vaccine in recurrent GB.

Trials	No.	Delivery routes	Target	Molecular characteristic	Toxicity	Phase	Results
ACTRN12609000338268	19	IV	Cytomegalovirus	NA	Minor adverse events; 1 pt with SAE for 3 d	I	mOS:403 d; 4 of 10pts with SD
	10	ID	Wilm’s tumor 1	NA	No grade 3–4 AEs	I	2pts with PR
NCT01130077	12	IH	EphA2, IL13Rα2, survivin	HLA-A2 + phenotype	No grade 3–4 AEs	I	mPFS:4.1 m; mOS:12.9 m
NCT02049489	20	ID	CD133	HLA-A2 + phenotype	NA	I	NA
NCT02078648	74	IV	Tumor-associated antigens	HLA-A2 + phenotype	NA	I/II	NA
NCT02808364	10	IV	Tumor-associated antigens	NA	NA	I	NA
NCT00293423	41	ID	HSPPC-96	NA	37pts grade 3-5 AEs	II	29.3%pts with OS:12 m; mOS:42.6w
NCT00846456	20	ID	DC vaccine	Cancer stem cell	Conventional grade 1-3AEs	I	mPFS:694 days vs mPFS:236 days
	17	ID	DC vaccine	NA	No grade 3-4 AEs	I	mPFS:1.9 m; mOS:10.9 m
	88	IH	Personalized peptide vaccination	HLA-A24 + phenotype	23pts with grade 3-4 AEs	III	mOS:8.4 m

*SAE* serious adverse event, *ID* intradermal injection, *IH* subcutaneous injection, *IC* infusions into tumor cavity, *IV* intravenous, *HSPPC* heat-shock protein peptide complex-96, *TNV* tumor necrotic volume.

### CAR-T cell immunotherapy in rGB

T cells modified to express a CAR are a promising therapeutic strategy that has achieved remarkable success in hematological malignancies^81–83^. The identification of highly restricted target antigens expressed on GB provides the foundation for the development of CAR-T cell therapy. Thus, related studies in rGB have been conducted. The first-in-human trial exploring CAR-engineered, autologous primary human CD8+ CTLs targeting IL-13Rα2 in rGB patients was conducted by Brown et al.^84,85^. They demonstrated that infusion of IL13-zetakine+ CTL clones into the resection cavity was well tolerated in all three rGB patients, and two of the patients exhibited transient antitumor responses. One of the responding patients showed reduced IL-13Rα2 expression within tumor tissue after CAR-T cell therapy, and the other patient appeared to have an increase in tumor necrotic volume at the site of CAR-T cell therapy. The same group also evaluated CAR-T cell therapy targeting IL-13Rα2 in an IDH1 wild-type, MGMT-nonmethylated rGB patient who had failed standard therapy. After CAR-T cell therapy, the patient’s intracranial and spinal tumors regressed. Additionally, the levels of cytokines and immune cells in the cerebrospinal fluid were obviously increased, demonstrating stimulation of the immune system manifested by specific trafficking and engraftment of T cells.

Ahmed et al.^86^ conducted a phase I study evaluating the immunoreactivity of HER2-specific CAR-T cell cranial cavity infusion therapy in 17 rGB patients (Table 3). The researchers used a second-generation CAR in this study, and no dose-limiting toxicity was observed. They demonstrated that the median OS time was 11.1 months from the first CAR-T cell infusion, and the disease control rate was 50%, with disease control times ranging from 8 weeks to 29 months. Among the patients, 3 had stable disease (SD) for 24 months to 29 months without any progression. This phase I trial demonstrated the feasibility and safety of peripheral injection of virus-specific CAR-T cells in rGB. Although CAR-T cells administered via this route do not undergo expansion in the blood, they have shown encouraging therapeutic effects.

**Table 3:** Clinical trials of CAR T-cell therapy in recurrent GB.

Trials	No.	Delivery routes	Target	Molecular characteristic	Toxicity	Phase	Results
NCT00730613	3	IC	IL13Rα2	NA	No grade 3 or higher	I	1 pt with reduced IL13Rα2 expression; 1 pt with an increment of TNV
NCT01109095	17	IV	Her2	NA	No dose-limiting toxic effects	I	1 pt with PR > 9 m; 7pts with SD for 8w to 29 m; 3pts with SD for 24-29 m; mOS: 24.5 m
NCT02442297	28	IC	Her2	NA	NA	I	NA
NCT02208362	1	IC then IV	IL13Rα2	MGMT non met IDH1 WT	No grade 3 or higher	I	Regression of all tumors; increment of cytokines and immune cells; clinical response for 7.5 m
NCT02209376	10	IV	EGFRvIII	MGMT non met	No off-tumor toxicity or cytokine release syndrome	I	1 pt with SD > 18 m; mOS:251days increment of inhibitory molecules and Treg
NCT02844062	20	IC	EGFRvIII	NA	NA	I	NA
NCT02937844	20	IV	Anti-PD-L1 CSR T cells	NA	NA	I	NA
NCT03170141	20	IV or IC	EGFRvIII	NA	NA	I	NA
NCT03389230	42	IT	HER2-CD3ζ-CD19t	NA	NA	I	NA
NCT03283631	24	IC	EGFRvIII	NA	NA	I	NA
NCT04045847	31	IC	CD147	NA	NA	I	NA
NCT04385173	12	IT	CD276	NA	NA	I	NA
NCT04077866	40	IT	CD276	NA	NA	I/II	NA
NCT04214392	36	IV	CD28-CD3ζ-CD19t	MMP2+	NA	I	NA
NCT04077866	100	IC	B7-H3	NA	No off-tumor toxicity	II	Effectively control tumor growth

*IC* infusions into tumor cavity, *IV* intravenous, *IT* intratumoral or intracerebroventricular injection.

The amino acid sequence resulting from a mutation in EGFRvIII produces a new glycine residue at the junction of exons 1 and 8 and results in immunogenic tumor-specific epitopes in the extracellular domain of epidermal growth factor receptor, which provides a theoretical basis for EGFRvIII-specific CAR-T cell therapy^87^. Furthermore, GB highly expresses the mutant tumor antigen EGFRvIII. O’Rourke et al.^88^ carried out the first-in-human study evaluating the immunoreactivity of EGFRvIII-specific CAR-T cells administered to 10 rGB patients by intravenous infusion. They found 1 patient had residual SD for over 18 months. In addition, researchers found that most GB patients had a specific loss or decreased expression of EGFRvIII in tumor tissue resected after CAR-T cell therapy^89^. However, pathological analysis of the tumor microenvironment confirmed that the adaptive immunosuppressive response was simultaneously activated with increased expression of inhibitory molecules (PD-L1, TGF-β, IDO, and IL-10) and infiltration of Tregs. From current phase I/II clinical trials, we conclude that CAR-T cell therapy has achieved only limited clinical efficacy in rGB patients and that the future of CAR-T cell therapy will depend on the effective recognition of tumor-specific antigens with sufficient and stable expression.

### Challenges and future directions for CAR-T cell therapy

#### Ameliorating the short lifespan of CAR-T cells

Although CAR-T cell therapy has made a breakthrough in the treatment of rGB, the persistence of CAR-T cells is a noteworthy issue that needs to be resolved to achieve durable clinical outcomes^90^. To address this issue, researchers have improved the drug delivery method of CAR-T cells from intravenous administration to infusion into tumor tissue^91^. Second, researchers have manufactured engineered T cells to express a costimulatory CAR and utilized CD28 end domains to construct CARs that can reduce the ex vivo expansion time of the T cells. Furthermore, a manufacturing platform using central memory T cells can be employed at the beginning of treatment. In addition, Long et al.^92^ demonstrated that a CAR based on CD28 end domains strengthened and accelerated T cell exhaustion, while a CAR based on 4-1BB end domains decelerated T cell exhaustion. In addition, Brown et al. optimized CAR-T cells with a 4-1BB costimulatory CAR and demonstrated that 4-1BB costimulatory CAR-T cells exhibited improved antitumor activity. Furthermore, the lifespan of the T cells was obviously ameliorated^93^.

#### Improving the poor infiltration by T cells

CAR-T cells cooperate with infiltrated T cells to attack tumor cells, but the quantity of infiltrated T cells is commonly sparse. To solve this issue, Adachi et al. engineered CAR-T cells to express IL-7 and CCL19 and found increased infiltration of DCs and T cells in tumor tissues, complete regression of tumors and prolonged mouse survival following engineered CAR-T cell therapy. Fortunately, they reported that both effector T cells and engineered CAR-T cells achieved memory responses against tumor cells. In addition, improved CAR-T cell persistence in tumors was demonstrated^94^. Therefore, if we can overcome the two major obstacles affecting efficacy in rGB, then CAR-T cell therapy combined with anti-PD-1/PD-L1 antibodies or other treatments will certainly achieve greater breakthroughs in rGB.

#### Overcoming tumor heterogeneity and antigen escape

The distribution of tumor-specific antigens, such as IL-13Rα2, Her2, and EGFRvIII, is heterogeneous in GB, and they are expressed to different degrees at different time points during CAR-T cell therapy^87,95^. Weller et al. found that the loss rate of the EGFRvIII antigen in the tumor tissue of GB patients who received rindopepimut treatment reached 57%. Moreover, the addition of rindopepimut to standard treatment with temozolomide did not improve the prognosis of patients, and the loss of the EGFRvIII antigen in the rindopepimut treatment group was not related to clinical benefit. The above results fully demonstrated that the regression of EGFRvIII+ tumor cells was accompanied by the progression of EGFRvIII− tumor cells, which offset the clinical benefits achieved with rindopepimut treatment targeting EGFRvIII+ tumor cells. Therefore, we have faced with the challenges of heterogeneity and antigen loss in the context of CAR-T cell therapy. It is very difficult to transform immunotherapy targeting a single antigen into durable clinical benefits. We still need to explore bispecific and trivalent CAR-T cell therapies in rGB patients.

#### Exploring potential combination strategies

Although the clinical activity of vaccination and CAR-T cell immunotherapy has been observed^84,85,96,97^, there is still much room for improvement in current immunotherapeutic strategies for rGB, and better results might be achieved by combining vaccination or CAR-T cell therapy with anti-PD-1/PD-L1 therapy to prevent immune resistance or escape, which can consolidate the immune response induced by vaccination. Tumor antigen heterogeneity and antigen escape are two prominent features of GB. Although DC vaccination targeting tumor peptides have demonstrated auspicious results in rGB patient treatment, Antonios et al.^98^ strongly suggested that anti-PD-1/PD-L1 therapy significantly augmented the adaptive immune response of tumor patients in response to vaccination. They found that DC vaccination in GB patients could upregulate PD-1 expression, while anti-PD-1 therapy following DC vaccination significantly augmented autologous tumor cell cytolysis. However, neither agent alone induced a survival benefit in mice with relatively large tumors. Moreover, this immunotherapeutic combination strategy had an immune memory effect that manifested as no tumor formation following reinoculation with the same glioma cells after combination therapy. In addition, there is strong evidence to support combining anti-PD-1/PD-L1 therapy with EGFRvIII-specific CAR-T cell therapy owing to breakthroughs in the treatment of rGB patients with EGFRvIII-specific CAR-T cell therapy^88^. An obvious clinical effect was demonstrated in refractory diffuse large B-cell lymphoma (DLBCL) after combination therapy with CD19 CAR-T cells and PD-1 checkpoint blockade^99^. In addition, John and colleagues confirmed that after specific stimulation with PD-L1+ tumor cells, transduced anti-Her-2 CD8+ T cells exhibited increased PD-1 expression. Simultaneously, the levels of activation and proliferation markers in anti-Her-2 T cells were significantly increased in the context of anti-PD-1 therapy^100^. In addition to EGFRvIII-specific CAR-T cell therapy combined with anti-PD-1/PD-L1 antibodies, CAR-T cell therapy targeting other GB-specific peptides, such as IL-13Rα2, HER2, or EphA2, combined with anti-PD-1/PD-L1 therapy is also a worthwhile therapeutic strategy.

In addition to PD-1, CTLA-4, TIM-3, and LAG-3 are inhibitory receptors expressed on the surface of T lymphocytes that downregulate T cell activity^101–104^. Anti-PD-1 therapy combined with anti-CTLA-4 therapy has demonstrated promising efficacy in preclinical GB mouse models^27,37^. In addition, one study found that LAG-3, an early marker of T cell exhaustion, is highly expressed in human glioblastoma specimens. Therefore, early treatment with an immune checkpoint inhibitor targeting LAG-3 was more effective than later treatment, and anti-LAG-3 antibodies could be combined with other immune checkpoint inhibitors to treat GB. The study showed that compared with the mice in the negative control group, the mice in the combined anti-LAG-3 (beginning on the 10th day after tumor implantation) and anti-PD-1 therapy group achieved a moderate survival benefit, while the mice in the combined anti-LAG-3 (beginning on the 7th day after tumor implantation) and anti-PD-1 therapy group achieved a significant survival benefit, indicating that anti-LAG-3 therapy can kill tumor cells more effectively in the early stage of tumorigenesis than in a later stage. More interestingly, the survival time of the mice in the LAG-3-knockdown combined with anti-PD-1 therapy group was significantly longer than that of the mice in the combined anti-LAG-3 and anti-PD-1 therapy group, indicating that LAG-3 plays an important role in the malignant progression of GB^105^. In addition, clinical trials exploring the clinical efficacy of combined targeting of CTLA-4, TIM-3, LAG-3, and PD-1/PD-L1 are underway. Furthermore, T lymphocytes also express costimulatory molecules, such as 4-1BB and OX40^106–108^. Nevertheless, the optimal sequence of combination immunotherapy should be validated to maximize the clinical effect. For instance, concurrent treatment with an agonist anti-OX40 antibody and an anti-PD-1 antibody could offset the antitumor effect of an agonist anti-OX40 antibody in a mammary cancer model^109^. Messenheimer et al. demonstrated that treatment with an agonist anti-OX40 antibody followed by an anti-PD-1 antibody could significantly augment antitumor efficacy^110^. The efficacy of these treatment regimens in rGB patients is worth further exploring.

Chemotherapy also synergizes with cancer DC vaccines to generate an effective antitumor effect. Many phase I and phase II studies have demonstrated the clinical efficacy of DC vaccination in GB patients^111–113^. Wheeler et al.^114^ demonstrated that vaccination combined with subsequent chemotherapy exhibited a significant survival benefit and prolonged time to tumor recurrence compared to vaccination or chemotherapy alone. Inspired by this research, we explored this combination strategy in rGB patients to augment vaccine-induced benefits and prolong survival.

**Fig. 3:**
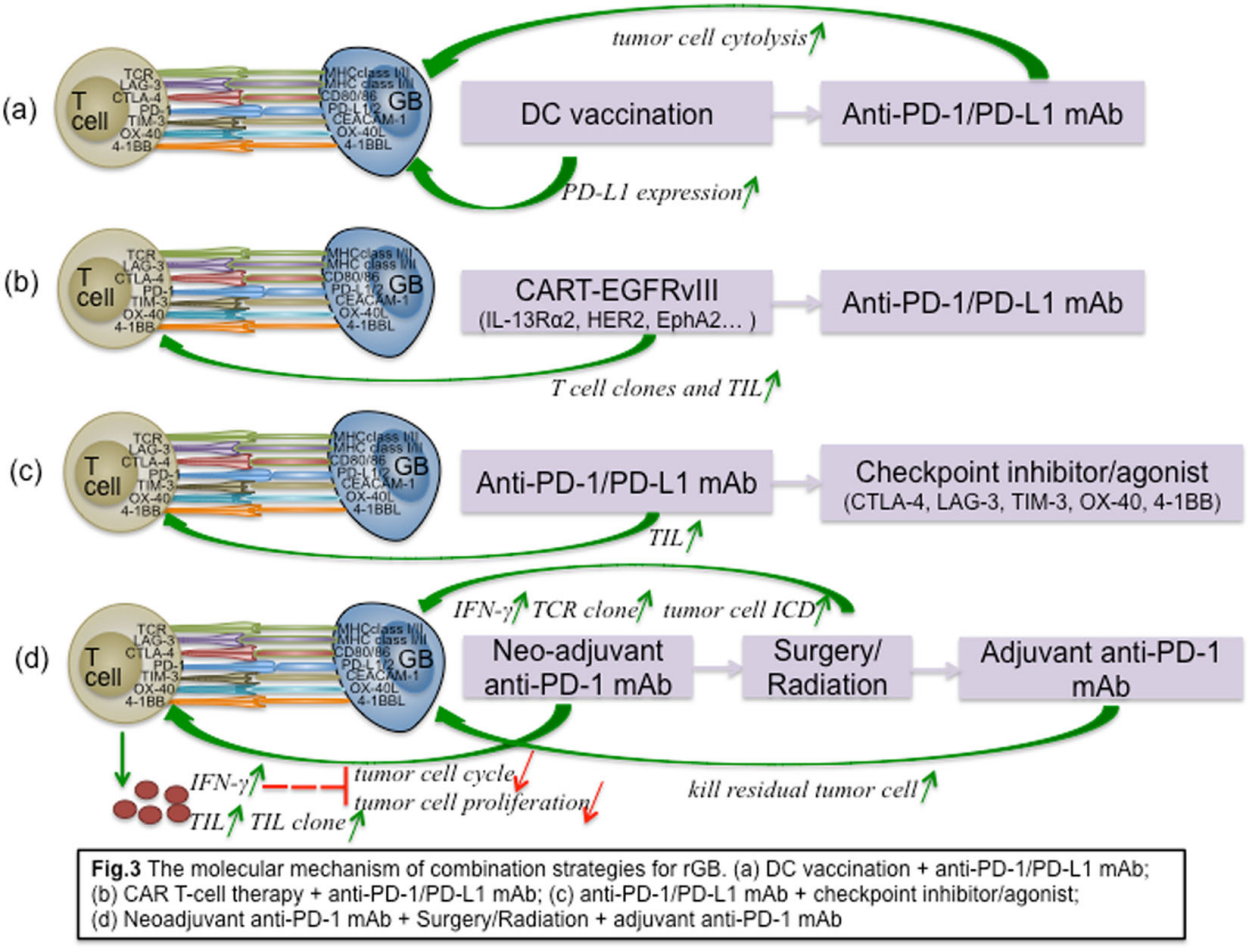
The molecular mechanism of combination strategies for rGB. **a** DC vaccination + anti-PD-1/PD-L1 mAb; **b** CAR T-cell therapy + anti-PD-1/PD-L1 mAb; **c** anti-PD-1/PD-L1 mAb + checkpoint inhibitor/agonist; **d** Neoadjuvant anti-PD-1 mAb + surgery/radiation + adjuvant anti-PD-1 mAb.

Patients with focal rGB located in an anatomical location that is easily resectable can undergo surgical resection. If rGB is located in an anatomical location that is difficult to surgically resect or if diffuse recurrence or multifocal recurrence occurs, reradiation is also an option^115^. Cloughesy and colleagues confirmed that the use of neoadjuvant anti-PD-1 therapy in rGB patients could effectively activate TIL function, produce an IFN-γ response, and inhibit the transcription of tumor cell cycle-related genes and tumor cell proliferation (Fig. 3). Surgical resection was then used to reduce the tumor burden while maintaining clonal tumor-specific T cell function. Adjuvant anti-PD-1 therapy following surgical resection could further kill residual tumor cells. The efficacy of this “sandwich treatment strategy” has been demonstrated in rGB patients^19^. Radiotherapy accurately kills tumor cells through high-energy physical rays while maximizing the protection of the surrounding normal brain tissue, which is a good alternative treatment for rGB patients who cannot undergo surgical resection. Moreover, studies have confirmed the synergistic effect of radiotherapy and anti-PD-1 therapy. Professor Formenti demonstrated that radiotherapy induces immunogenic cell death (ICD), IFN-γ release and increased TCR diversity, thereby enhancing the clinical effect of anti-PD-1 therapy on tumors^116,117^. Therefore, the clinical practices of neoadjuvant anti-PD-1 therapy + surgical resection + adjuvant anti-PD-1 therapy and neoadjuvant anti-PD-1 therapy + radiotherapy + adjuvant anti-PD-1 therapy are also worth exploring as treatments for rGB patients.

## Conclusion

GB is a tumor with highly malignant biological behavior, and more than 90% of GB patients will experience recurrence and progression. The mOS time of rGB is only 6-11 months owing to the lack of effective treatments. Although immunotherapy has achieved breakthroughs in treating rGB patients, many limitations need to be taken into consideration.

GB is a well-known “immunologically cold tumor” and its tumor microenvironment contains very few cytotoxic T lymphocytes. Under these circumstances, even if the PD-1/PD-L1 pathway is blocked, patients cannot mobilize enough effector T cells to kill their tumor cells. It is likely that other immune sites in GB play relatively important and decisive roles. Furthermore, in the course of anti-PD-1 treatment for rGB, the occurrence of adaptive resistance is also an important reason for treatment failure, including exhaustion of cytotoxic T lymphocytes resulting from the emergence of coinhibitory molecules during treatment^62^. Tumor heterogeneity leads to the killing of some tumor cells but survival and expansion of subclonal cells that have no response to anti-PD-1 treatment^118^.

Moreover, the blood–brain barrier (BBB) makes it difficult for drugs and lymphocytes to infiltrate into tumor tissues, which usually leads to treatment failure. Some studies^119^ have shown that CAR-T cell therapy can increase the number of effector lymphocytes in tumor tissue, thus improving the immune response and curing GB. However, this approach is not suitable for patients with primary GB, especially those who have not undergone surgery, because an increased number of lymphocytes can also increase the tumor volume in a short time, which leads to an increase in intracranial pressure that results in disease aggravation. Nevertheless, for postoperative GB patients and rGB patients, tumor load reduction can provide enough space for increased lymphocyte infiltration.

However, CAR-T cell treatment also has shortcomings. First, how many lymphocytes with cytotoxic activity can pass through the BBB? This needs further experimental verification. If CAR-T cells are injected into the tumor cavity to improve BBB permeability, the safety of this method must be considered. Second, the heterogeneity of rGB prevents CAR-T cells from completely eliminating tumors, which lays the groundwork for the recurrence of surviving tumor cells. Moreover, as mentioned above, CAR-T cells exhibit a short lifespan and poor infiltration, which directly affects their cytotoxic effect on rGB. How to identify more effective CAR-T cell targets for the treatment of rGB is also important.

For tumor vaccines used to treat rGB, we are also concerned about the lack of effective therapeutic targets considering that GB usually has a very low tumor mutational burden (TMB) and immunogenicity, the problem of activated cytotoxic cell transit through the BBB, and the high heterogeneity of recurrent tumors, which usually lead to tumor treatment failure.

However, we still believe that immunotherapy can be used as an alternative treatment or a therapeutic option for rGB. Cloughesy et al.^21^ confirmed that neoadjuvant anti-PD-1 treatment achieved benefits in patients with rGB. Although the sample size of this randomized controlled trial was small, with only 35 people enrolled, the results are encouraging. In addition, we also found that the prognosis of patients with intracranial infection after tumor resection was better than that of uninfected patients, suggesting that the increase in intracranial lymphocytes (whether mononuclear cytotoxic cells or polynuclear neutrophils) may lead to tumor elimination through an immune pathway.

Therefore, immunotherapy for rGB still has the following problems. First, selecting the optimal sequence for immunotherapy administration is important for achieving good efficacy. Cloughesy et al.^21^ confirmed that neoadjuvant anti-PD-1 therapy followed by surgery and adjuvant anti-PD-1 therapy could effectively activate local and systemic immune responses and significantly improve the OS of rGB patients. Second, selecting optimal combination strategies to overcome immunosuppressive factors is also challenging. Approaches using anti-PD-1/PD-L1 antibodies combined with antibodies targeting CTLA-4, TIM-3, LAG-3, 4-1BB, or OX-40 are under exploration^120^. Furthermore, anti-PD-1/PD-L1 therapy combined with tumor-specific peptide vaccination or CAR-T cell therapy^121,122^ is also worth exploring. Third, exploring effective predictive biomarkers of clinical efficacy is a pivotal issue to avoid economic waste and treatment delay. GB has low immunogenicity with a low TMB and low neoantigen levels^123^. GB patients with MMR protein deficiency or POLE mutations have been reported to have a high treatment response rate^124,125^. Attempts to identify predictive biomarkers of clinical efficacy in the treatment of rGB will undoubtedly face challenges. Fourth, engineering techniques to prolong the CAR-T cell lifespan and increase T cell infiltration need to be further considered to strengthen T cell function. Fifth approaches to open the BBB, improve drug permeability, and increase effector tumor-specific cytotoxic cell levels are also very important for enhancing the effectiveness of treatments.

In conclusion, there are still many doubts and uncertainties regarding the use of immunotherapy for the treatment of GB, especially rGB. Issues related to the approach, timing, patient population, and combinations with other treatments will need to be solved.

## References

[1] Darlix A (2017). Epidemiology for primary brain tumors: a nationwide population-based study. J. Neurooncol..

[2] Ostrom QT (2015). CBTRUS statistical report: primary brain and central nervous system tumors diagnosed in the United States in 2008–2012. Neuro Oncol..

[3] Koshy M (2012). Improved survival time trends for glioblastoma using the SEER 17 population-based registries. J. Neurooncol..

[4] Stupp R (2005). Mirimanoff. Radiotherapy plus concomitant and adjuvant temozolomide for glioblastoma. N. Engl. J. Med..

[5] Stupp R (2009). Effects of radiotherapy with concomitant and adjuvant temozolomide versus radiotherapy alone on survival in glioblastoma in a randomised phase III study: 5-year analysis of the EORTC- NCIC trial. Lancet Oncol..

[6] Hottinger AF, Pacheco P, Stupp R (2016). Tumor treating fields: a novel treatment modality and its use in brain tumors. Neuro Oncol..

[7] Lamborn KR (2008). Progression-free survival: an important end point in evaluating therapy for recurrent high-grade gliomas. Neuro Oncol..

[8] Wu W (2010). Joint NCCTG and NABTC prognostic factors analysis for high-grade recurrent glioma. Neuro Oncol..

[9] Clarke JL (2011). Is surgery at progression a prognostic marker for improved 6-month progression-free survival or overall survival for patients with recurrent glioblastoma?. Neuro Oncol..

[10] Batchelor TT (2010). Phase II study of cediranib, an oral pan-vascular endothelial growth factor receptor tyrosine kinase inhibitor, in patients with recurrent glioblastoma. J. Clin. Oncol..

[11] Scott BJ (2010). Bevacizumab salvage therapy following progression in high-grade gliomas patients treated with VEGF receptor tyrosine kinase inhibitors. Neuro Oncol..

[12] Friedman HS (2009). Bevacizumab alone and in combi- nation with irinotecan in recurrent glioblastoma. J. Clin. Oncol..

[13] Paez-Ribes M (2009). Antiangiogenic therapy elicits malignant progression of tumors to increased local invasion and distant metastasis. Cancer Cell.

[14] Dyck L, Mills KHG (2017). Immune checkpoints and their inhibition in cancer and infectious diseases. Eur. J. Immunol..

[15] Ledford H, Else H, Warren M (2018). Cancer immunologists scoop medicine Nobel prize. Nature.

[16] Rotte (2018). Noble committee honors tumor immunologists. J. Exp. Clin. Cancer Res..

[17] Shen CR, Chen YS (2015). Immune checkpoint blockade therapy: the 2014 Tang prize in biopharmaceutical science. Biomed. J..

[18] Xu F, Jin T, Zhu Y, Dai C (2018). Immune checkpoint therapy in liver cancer. J. Exp. Clin. Cancer Res..

[19] Faghfuri E, Faramarzi MA, Nikfar S, Abdollahi M (2015). Nivolumab and pembrolizumab as immune-modulating monoclonal antibodies targeting the PD-1 receptor to treat melanoma. Expert Rev. Anticancer Ther..

[20] Filley AC, Henriquez M, Dey M (2017). Recurrent glioma clinical trial, checkmate-143: the game is not over yet. Oncotarget.

[21] Cloughesy TF (2019). Neoadjuvant anti-PD-1 immunotherapy promotes a survival benefit with intratumoral and systemic immune responses in recurrent glioblastoma. Nat. Med..

[22] Verhaak RG (2010). Integrated genomic analysis identifies clinically relevant subtypes of glioblastoma characterized by abnormalities in PDGFRA, IDH1, EGFR, and NF1. Cancer Cell.

[23] Li R (2015). Comprehensive portrait of recurrent glioblastoma multiforme in molecular and clinical characteristics. Oncotarget.

[24] Rahman M (2018). Analysis of immunobiologic markers in primary and recurrent glioblastoma. J. Neuro-Oncol..

[25] Berghoff AS (2017). Correlation of immune phenotype with IDH mutation in diffuse glioma. Neuro-Oncol..

[26] Friedman HS (2009). Bevacizumab alone and in combination with irinotecan in recurrent glioblastoma. J. Clin. Oncol..

[27] Rutledge WC (2013). Tumor-infiltrating lymphocytes in glioblastoma are associated with specific genomic alterations and related to transcriptional class. Clin. Cancer Res..

[28] Kmiecik J (2013). Elevated CD3+ and CD8+ tumor-infiltrating immune cells correlate with prolonged survival in glioblastoma patients despite integrated immunosuppressive mechanisms in the tumor microenvironment and at the systemic level. Neuro Immunol..

[29] Chen D (2020). Absolute lymphocyte count predicts abscopal responses and outcomes in patients receiving combined immunotherapy and radiotherapy. Int. J. Radiat. Oncol. Biol. Phys..

[30] Antonios JP (2017). Detection of immune responses after immunotherapy in glioblastoma using PET and MRI. Proc. Natl Acad. Sci. USA.

[31] Berghoff AS (2015). Programmed death ligand 1 expression and tumor-infiltrating lymphocytes in glioblastoma. Neuro Oncol..

[32] Zeng J (2013). Anti-PD-1 blockade and stereotactic radiation produce long-term survival in mice with intracranial gliomas. Int. J. Radiat. Oncol. Biol. Phys..

[33] Huang BY (2015). The PD-1/B7-H1 pathway modulates the natural killer cells versus mouse glioma stem cells. PLoS ONE.

[34] Gordon S, Martinez FO (2010). Alternative activation of macrophages: mechanism and functions. Immunity.

[35] Saha D, Martuza RL, Rabkin SD (2017). Macrophage polarization contributes to glioblastoma eradication by combination immunovirotherapy and immune checkpoint blockade. Cancer Cell.

[36] Simonelli M (2018). Checkpoint inhibitors as treatment for malignant gliomas: “A long way to the top”. Cancer Treat. Rev..

[37] Wang X (2019). Challenges and potential of PD-1/PD-L1 checkpoint blockade immunotherapy for glioblastoma. J. Exp. Clin. Cancer Res..

[38] Wei J (2010). Glioblastoma cancer-initiating cells inhibit T-cell proliferation and effector responses by the signal transducers and activators of transcription 3 pathway. Mol. Cancer Ther..

[39] Zhang J (2012). A dialog between glioma and microglia that promotes tumor invasiveness through the CCL2/CCR2/interleukin-6 axis. Carcinogenesis.

[40] Kohanbash G (2013). GM-CSF promotes the immunosuppressive activity of glioma-infiltrating myeloid cells through interleukin-4 receptor-α. Cancer Res..

[41] Yang I, Han SJ, Kaur G, Crane C, Parsa AT (2010). The role of microglia in central nervous system immunity and glioma immunology. J. Clin. Neurosci..

[42] Kuratsu J (1993). Quantitative study of monocyte chemoattractant protein-1 (MCP-1) in cerebrospinal fluid and cyst fluid from patients with malignant glioma. J. Natl Cancer Inst..

[43] Thornton AM, Shevach EMCD4 (1998). CD25+ immunoreg- ulatory T cells suppress polyclonal T cell activation in vitro by inhibiting interleukin 2 production. J. Exp. Med.

[44] Camara NO, Sebille F, Lechler RI (2003). Human CD4+CD25+ regulatory cells have marked and sustained effects on CD8+ T cell activation. Eur. J. Immunol..

[45] Piccirillo CA, Shevach EM (2001). Cutting edge: control of CD8+ T cell activation by CD4+CD25+immunoregulatory cells. J. Immunol..

[46] Dieckmann D, Bruett CH, Ploettner H, Lutz MB, Schuler G (2002). Human CD4(+)CD25(+) regulatory, contact- dependent T cells induce interleukin 10-producing, contact-independent type 1-like regulatory T cells. J. Exp. Med..

[47] Wintterle S (2003). Expression of the B7-related molecule B7-H1 by glioma cells: a potential mechanism of immune paralysis. Cancer Res..

[48] Wainwright DA (2012). IDO expression in brain tumors increases the recruitment of regulatory T cells and negatively impacts survival. Clin. Cancer Res..

[49] Abou-Ghazal M (2008). The incidence, correlation with tumor-infiltrating inflammation, and prognosis of phosphorylated STAT3 expression in human gliomas. Clin. Cancer Res..

[50] Facoetti A (2005). Human leukocyte antigen and antigen processing machinery compo- nent defects in astrocytic tumors. Clin. Cancer Res..

[51] Anderson RC (2007). Lack of B7 expression, not human leukocyte antigen expression, facilitates immune evasion by human malignant gliomas. Neurosurgery.

[52] Parsa AT (2007). Loss of tumor suppressor PTEN function increases B7-H1 expression and immunoresistance in glioma. Nat. Med..

[53] Dai S, Jia R, Zhang X, Fang Q, Huang L (2014). The PD-1/PD-Ls pathway and autoimmune diseases. Cell. Immunol..

[54] Victor TS (2015). Radiation and dual checkpoint blockade activates non-redundant immune mechanisms in cancer. Nature.

[55] Gettinger S, Herbst RS (2014). B7-H1/PD-1 blockade therapy in non-small cell lung cancer: current status and future direction. Cancer J..

[56] Topalian SL (2012). Safety, activity, and immune correlates of anti-PD-1 antibody in cancer. N. Engl. J. Med..

[57] Taube JM (2014). Association of PD-1, PD-1 ligands, and other features of the tumor immune microenvironment with response to anti-PD-1 therapy. Clin. Cancer Res..

[58] Weber JS (2015). Nivolumab versus chemotherapy in patients with advanced melanoma who progressed after anti-CTLA-4 treatment (CheckMate 037): a randomised, controlled, open-label, phase 3 trial. Lancet Oncol..

[59] Zhao J (2019). Immune and genomic correlates of response to anti-PD-1 immunotherapy in glioblastoma. Nat. Med..

[60] Wherry EJ, Kurachi M (2015). Molecular and cellular insights into T cell exhaustion. Nat. Rev. Immunol..

[61] Sakuishi K (2010). Targeting Tim-3 and PD-1 pathways to reverse T cell exhaustion and restore anti-tumor immunity. J. Exp. Med..

[62] Koyama S (2016). Adaptive resistance to therapeutic PD-1 blockade is associated with upregulation of alternative immune checkpoints. Nat. Commun..

[63] Kjaergaard J (2000). Therapeutic efficacy of OX-40 receptor antibody depends on tumor immunogenicity and anatomic site of tumor growth. Cancer Res..

[64] Fecci PE (2007). SystemicCTLA-4blockade ameliorates glioma-induced changes to the CD4+ T cell compartment without affecting regulatory T-cell function. Clin. Cancer Res..

[65] Schalper KA (2019). Neoadjuvant nivolumab modifies the tumor immune microenvironment in resectable glioblastoma. Nat. Med..

[66] Ahmed N (2010). HER2-specific T cells target primary glioblastoma stem cells and induce regression of autologous experimental tumors. Clin. Cancer Res..

[67] Heimberger AB (2008). Immunological responses in a patient with glioblastoma multiforme treated with sequential courses of temozolomide and immunotherapy: case study. Neuro Oncol..

[68] Saikali S (2007). Expression of nine tumour antigens in a series of human glioblastoma multiforme: interest of EGFRvIII, IL- 13Ralpha2, gp100 and TRP-2 for immunotherapy. J. Neurooncol..

[69] Sampson JH, Archer GE, Mitchell DA, Heimberger AB, Bigner DD (2008). Tumor-specific immunotherapy targeting the EGFRvIII mutation in patients with malignant glioma. Semin. Immunol..

[70] Zhu X (2015). Patient-derived glioblastoma stem cells are killed by CD133-specific CAR T cells but induce the T cell aging marker CD57. Oncotarget.

[71] Desjardins A (2018). Recurrent glioblastoma treated with recombinant poliovirus. N. Engl. J. Med..

[72] Bloch O (2014). Heat-shock protein peptide complex-96 vaccination for recurrent glioblastoma: a phase II, single-arm trial. Neuro Oncol..

[73] Rudnick JD (2020). A phase I trial of surgical resection with Gliadel Wafer placement followed by vaccination with dendritic cells pulsed with tumor lysate for patients with malignant glioma. J. Clin. Neurosci..

[74] Sakai K (2015). Dendritic cell–based immunotherapy targeting Wilms’ tumor 1 in patients with recurrent malignant glioma. J. Neurosurg..

[75] Terasaki M (2011). Phase I trial of a personalized peptide vaccine for patients positive for human leukocyte antigen—A24 with recurrent or progressive glioblastoma multiforme. J. Clin. Oncol..

[76] De Vleeschouwer S (2008). Postoperative adjuvant dendritic cell-based immunotherapy in patients with relapsed glioblastoma multiforme. Clin. Cancer Res..

[77] Rutkowski S (2004). Surgery and adjuvant dendritic cell-based tumour vaccination for patients with relapsed malignant glioma, a feasibility study. Br. J. Cancer.

[78] Shah AH, Bregy A, Heros DO, Komotar RJ, Goldberg J (2013). Dendritic cell vaccine for recurrent high-grade gliomas in pediatric and adult subjects: Clinical Trial Protocol. Neurosurgery.

[79] Okada H (2011). Induction of CD8+ T-cell responses against novel glioma-associated antigen peptides and clinical activity by vaccinations with α-type 1 polarized dendritic cells and polyinosinic-polycytidylic acid stabilized by lysine and carboxymethylcellulose in patients with recurrent malignant glioma. J. Clin. Oncol..

[80] Narita Y (2019). A randomized, double-blind, phase III trial of personalized peptide vaccination for recurrent glioblastoma. Neuro Oncol..

[81] Porter DL, Levine BL, Kalos M, Bagg A, June CH (2011). Chimeric antigen receptor-modified T cells in chronic lymphoid leukemia. N. Engl. J. Med.

[82] Grupp SA (2013). Chimeric antigen receptor-modified T cells for acute lymphoid leukemia. N. Engl. J. Med..

[83] Maude SL (2014). Chimeric antigen receptor T cells for sustained remissions in leukemia. N. Engl. J. Med..

[84] Brown CE (2015). Bioactivity and safety of IL13Rα2-redirected chimeric antigen receptor CD8+ T cells in patients with recurrent glioblastoma. Clin. Cancer Res..

[85] Brown CE (2016). Regression of glioblastoma after chimeric antigen receptor T-cell therapy. N. Engl. J. Med..

[86] Ahmed N (2017). HER2-specific chimeric antigen receptor-modified virus-specific T cells for progressive glioblastoma: a phase 1 dose-escalation trial. JAMA Oncol..

[87] Weller M (2017). ACT IV trial investigators. Rindopepimut with temozolomide for patients with newly diagnosed, EGFRvIII-expressing glioblastoma (ACT IV): a randomised, double-blind, international phase 3 trial. Lancet Oncol..

[88] O’Rourke DM (2017). A single dose of peripherally infused EGFRvIII- directed CAR T cells mediates antigen loss and induces adaptive resistance in patients with recurrent glioblastoma. Sci. Transl. Med..

[89] Felsberg J (2017). Prognostic role of epidermal growth factor receptor variant III (EGFRvIII) positivity in EGFR- amplified primary and recurrent glioblastomas. Clin. Cancer Res..

[90] Brown CE (2012). Stem-like tumor-initiating cells isolated from IL13Ralpha2 expressing gliomas are targeted and killed by IL13- zetakine-redirected T cells. Clin. Cancer Res..

[91] Rodriguez A, Brown C, Badie B (2017). Chimeric antigen receptor T-cell therapy for glioblastoma. Transl. Res..

[92] Long AH (2015). 4-1BB costimulation ameliorates T cell exhaustion induced by tonic signaling of chimeric antigen receptors. Nat. Med..

[93] Brown CE (2018). Optimization of IL13Ra2-targeted chimeric antigen receptor T cells for improved anti-tumor efficacy against glioblastoma. Mol. Ther..

[94] Adachi K (2018). IL-7 and CCL19 expression in CAR-T cells improves immune cell infiltration and CAR-T cellsurvival in the tumor. Nat. Biotechnol..

[95] Eskilsson E (2018). EGFR heterogeneity and implications for therapeutic intervention in glioblastoma. Neuro Oncol..

[96] Akiyama Y (2012). α type-1 polarized dendritic cell-based vaccination in recurrent high-grade glioma: a phase I clinical trial. BMC Cancer.

[97] Pellegatta S (2013). The natural killer cell response and tumor debulking are associated with prolonged survival in recurrent glioblastoma patients receiving dendritic cells loaded with autologous tumor lysates. Oncoimmunology.

[98] Antonios JP (2016). PD-1 blockade enhances the vaccination-induced immune response in glioma. JCI Insight.

[99] Chong EA (2017). PD-1 blockade modulates chimeric antigen receptor (CAR)-modified T cells: refueling the CAR. Blood.

[100] John LB (2013). Anti-PD-1 antibody therapy potently enhances the eradication of established tumors by gene-modified T cells. Clin. Cancer Res..

[101] Maes W, Van, Gool SW (2011). Experimental immunotherapy for malignant glioma: lessons from two decades of research in the GL261 model. Cancer Immunol. Immunother..

[102] Han S (2014). Tim-3 on peripheral CD4+ and CD8+ T cells is involved in the development of glioma. DNA Cell Biol..

[103] Li G (2017). Molecular and clinical characterization of TIM- 3 in glioma through 1,024 samples. Oncoimmunology.

[104] Granier C (2017). Mechanisms of action and rationale for the use of checkpoint inhibitors in cancer. ESMO Open.

[105] Harris-Bookman S (2018). Expression of LAG-3 and efficacy of combination treatment with anti-LAG-3 and anti-PD-1 monoclonal antibodies in glioblastoma. Int. J. Cancer.

[106] Shindo Y (2015). Combination immunotherapy with 4-1BB activation and PD-1 blockade enhances antitumor efficacy in a mouse model of subcutaneous tumor. Anticancer Res..

[107] Croft M, So T, Duan W, Soroosh P (2009). The significance of OX40 and OX40L to T-cell biology and immune disease. Immunol. Rev..

[108] Munks MW, Mourich DV, Mittler RS, Weinberg AD, Hill AB (2004). 4-1BB and OX40 stimulation enhance CD8 and CD4 T- cell responses to a DNA prime, poxvirus boost vaccine. Immunology.

[109] Messenheimer DJ (2017). Timing of PD-1 blockade is critical to effective combination immunotherapy with Anti-OX40. Clin. Cancer Res..

[110] Shrimali RK (2017). Concurrent PD-1 blockade negates the effects of OX40 agonist antibody in combination immunotherapy through inducing T-cell apoptosis. Cancer Immunol. Res..

[111] Sampson JH (2010). Immunologic escape after prolonged progression-free survival with epidermal growth factor receptor variant III peptide vaccination in patients with newly diagnosed glioblastoma. J. Clin. Oncol..

[112] Chang CN (2011). A phase I/II clinical trial investigating the adverse and therapeutic effects of a postoperative autologous dendritic cell tumor vaccine in patients with malignant glioma. J. Clin. Neurosci..

[113] Jie X (2012). Clinical application of a dendritic cell vaccine raised against heat-shocked glioblastoma. Cell Biochem. Biophys..

[114] Wheeler CJ, Das A, Liu G, Yu JS, Black KL (2004). Clinical responsiveness of glioblastoma multiforme to chemotherapy after vaccination. Clin. Cancer Res..

[115] Nabors LB (2017). NCCN guidelines insights: central nervous system cancer, Version 1.2017. J. Natl Compr. Cancer Netw..

[116] Formenti SC, Demaria S (2013). Combining radiotherapy and cancer immunotherapy: a paradigm shift. J. Natl Cancer Inst..

[117] Teng F, Kong L, Meng X, Yang J, Yu J (2015). Radiotherapy combined with immune checkpoint blockade immunotherapy: achievements and challenges. Cancer Lett..

[118] Woroniecka K (2018). T cell exhaustion signatures vary with tumor type and are severe in glioblastoma. Clin. Cancer Res..

[119] Bagley SJ, Desai AS, Linette GP, June CH, O’Rourke. DM (2018). CAR T-cell therapy for glioblastoma: recent clinical advances and future challenges. Neuro Oncol..

[120] Omuro A (2018). Nivolumab with or without ipilimumab in patients with recurrent glioblastoma: results from exploratory phase I cohorts of CheckMate 143. Neuro Oncol..

[121] Schuessler A (2014). Autologous T-cell therapy for cytomegalovirus as a consolidative treatment for recurrent glioblastoma. Cancer Res..

[122] Nehama D (2019). B7-H3-redirected chimeric antigen receptor T cells target glioblastoma and neurospheres. EBiomedicine.

[123] Schumacher TN, Schreiber RD (2015). Neoantigens in cancer immunotherapy. Science.

[124] Bouffet E (2016). Immune checkpoint inhibition for hypermutant glioblastoma multiforme resulting from germline biallelic mismatch repair deficiency. J. Clin. Oncol..

[125] Johanns TM (2016). Immunogenomics of hypermutated glioblastoma: a patient with germline POLE deficiency treated with checkpoint blockade immunotherapy. Cancer Discov..

